# Implementation of repeat HIV testing during pregnancy in southwestern Kenya: progress and missed opportunities

**DOI:** 10.1002/jia2.25036

**Published:** 2017-12-13

**Authors:** Anna J Rogers, Eliud Akama, Elly Weke, Justin Blackburn, George Owino, Elizabeth A Bukusi, Patrick Oyaro, Zachary A Kwena, Craig R Cohen, Janet M Turan

**Affiliations:** ^1^ Department of Health Care Organization and Policy University of Alabama at Birmingham School of Public Health Birmingham AL USA; ^2^ Centre for Microbiology Research Kenya Medical Research Institute Nairobi Kenya; ^3^ Department of Global Health University of Washington School of Public Health Seattle WA USA; ^4^ Department of Obstetrics, Gynecology& Reproductive Sciences University of California San Francisco CA USA

**Keywords:** PMTCT, implementation science, repeat HIV testing, pregnancy, Kenya

## Abstract

**Introduction:**

Repeat HIV testing during the late antenatal period is crucial to identify and initiate treatment for pregnant women with incident HIV infection to prevent perinatal HIV transmission and keep mothers alive. In 2012, the Kenya Ministry of Health adopted international guidelines suggesting that pregnant women be offered retesting three months after an initial negative HIV test. Our objectives were to determine the current rate of antenatal repeat HIV testing; identify successes, missed opportunities and factors associated with retesting; and estimate the incidence of HIV during pregnancy.

**Methods:**

Retrospective analysis of longitudinal data was conducted for a cohort of 2145 women attending antenatal care clinic at a large district hospital in southwestern Kenya. Data were abstracted from registers for all women who attended the clinic from the years 2011 to 2014.

**Results:**

Although 90.2% of women first came to clinic prior to their third trimester and 27.5% had at least four clinic visits, 58.0% of all women went to delivery without a retest. Missed opportunities for retesting included not returning to clinic at all, not returning when eligible, or late gestational age (>28 weeks) at first clinic visit making them ineligible for retesting (accounting for 14.2%, 26.8% and 9.6% of all clinic attendees respectively); and failure to be retested even when eligible at one or more visits (accounting for 73.2% of eligible returnees). Being unmarried and aged 20 or younger was associated with an increase in mean gestational age of first visit by 2.52 weeks (95% CI: 1.56, 3.48) and a 2.59 increased odds (95% CI: 1.90, 3.54) of failing to return to clinic, compared to those who were married and over 20 years of age. On retest, two women tested HIV positive, suggesting an incidence rate of 4.4 per 100 person‐years. After adjusting for potential confounders, only later year of last menstrual period (2013 vs. 2012 and 2011) was associated with retesting.

**Conclusions:**

Adoption of retesting guidelines in 2012 appears to have successfully increased retesting rates, but missed opportunities to identify incident HIV infection during pregnancy may contribute to continuing high rates of perinatal HIV transmission in southwestern Kenya.

## 1 Introduction

Pregnant women with chronic HIV infection are increasingly identified as seropositive and being placed on antiretroviral treatment (ART) at first antenatal care (ANC) visit due to the expansion of HIV testing into ANC settings and the adoption of policies suggesting immediate and lifelong initiation of ART for pregnant women (“Option B+”) 1, 2. Thus, pregnant women who initially test HIV negative in ANC and then subsequently experience HIV seroconversion during the perinatal period are likely to be contributing to a growing proportion of mother‐to‐child transmission (MTCT) events 3. This is particularly true because acute HIV infection is associated with elevated viral loads that increase risk of transmission during pregnancy, delivery and through breastfeeding 4, 5.

Repeat HIV testing during pregnancy allows women who have seroconverted since first antenatal test to be aware of their HIV status and initiate ART for their own sake, as well as to prevent MTCT 2. Infants born to mothers of known HIV‐positive status are often more closely followed as HIV‐exposed infants: they are given HIV prophylaxis at delivery and for a period of time after birth, tested through early infant diagnosis programmes, and immediately initiated on ART if found to be infected with HIV 6, 7.

In Kenya, there are roughly 1.5 million pregnancies and 87,000 HIV‐positive pregnant women per annum 8. The MTCT rate was estimated in 2012 to be 15%, accounting for 13,000 new childhood infections in Kenya annually 9, 10. In 2012, the Kenya Ministry of Health adopted international guidelines recommending that repeat HIV testing be offered three months after an initial negative HIV test result in early pregnancy 11. While one study in Kenya found the acceptability of provider‐initiated retesting in late pregnancy to be 93.5% 12, only one known study from Zambia has reported the retesting rate in a non‐intervention setting to be 24.5% among eligible pregnant women 13. In addition, little is known about gaps in implementation of repeat HIV testing and the factors associated with a lack of retesting, thus making it challenging to address the deficits.

The purpose of this paper was to determine the current rate of antenatal repeat HIV testing; identify successes, missed opportunities and factors associated with retesting; and estimate the incidence of HIV during pregnancy at a large hospital in southwestern Kenya.

## 2 Methods

### 2.1 Setting and Context

This study was conducted at a large government hospital in rural southwestern Kenya, an area of the country with the highest HIV prevalence at 15.1% 14. The facility is one of three district hospitals in the county and has a large patient volume comprising of primarily low‐income clients. The Kenya AIDS Indicator Survey 2012 found that for Kenya overall, 95.4% of reproductive‐aged women attended ANC clinic during pregnancy, 93.1% of whom were tested for HIV at least once during their last pregnancy 15. In the study setting, the Kenya Ministry of Health facility was supported by Family AIDS Care and Education Services (FACES), a PEPFAR‐funded programme that provides integrated HIV and ANC care 16.

### 2.2 Study Design

In early 2015, longitudinal antenatal record data for the full pregnancy were abstracted from paper ANC registers for all women attending the antenatal clinic of the hospital in the years 2011 to 2014. Data for pregnant women were excluded if they had their last menstrual period (LMP) in the year 2014, since these women may not have records for their entire pregnancy included in the dataset, or if their first antenatal visit was at a different clinic, since it would constitute missing information on gestational age at first ANC visit and HIV test result. Ethical approval was given by the Kenya Medical Research Institute Scientific and Ethical Review Unit (SERU) and the University of Alabama at Birmingham Institutional Review Board. As the data were gathered as a part of routine medical care and de‐identified after linkage, individual patient consent was not solicited as a part of this study.

### 2.3 Variable Definitions

Women were coded as having had an initial HIV test if their records noted that they tested HIV‐negative or ‐positive on their first ANC clinic visit, and they were not an individual with a “known HIV‐positive” status at the start of the visit. Women were eligible for a repeat HIV test if they visited the ANC clinic again at least 12 weeks after their initial visit with an HIV‐negative test result. Women were coded as having been retested if records indicated an HIV‐negative or ‐positive test on an eligible re‐visit; otherwise a missed opportunity was noted accordingly. Women were ineligible for a retest if they had previously tested positive for HIV or if they did not come early enough for their first clinic visit (i.e. ≤28 weeks gestational age) to have another HIV test during the current pregnancy, as per national guidelines.

Seroconversion during pregnancy was defined as having an HIV‐negative result at the initial HIV test, and an HIV‐positive result on retest at least 12 weeks later. Village distance from the hospital was estimated by utilizing the expertise of local facility transport staff, who deliver supplies such as medicines and biological specimens between government health facilities, to determine the distance between women's village locations as listed in the ANC register and the hospital. Pregnancy cohorts were defined by the year of their LMP.

### 2.4 Data Analysis

Data that had been entered into an electronic database were cleaned and analysed in SAS Version 9.4 (SAS Institute Inc., Cary, NC, USA). Bivariate and multivariable analyses were conducted using linear and logistic regression, along with linear trend analyses, to assess the statistical association of patient demographic variables with process measures, including gestational age at first visit and returning to antenatal clinic, as well as outcome measures, such as getting a repeat HIV test 17. Specifically, to understand the characteristics of women who received repeat HIV testing as compared to those who did not, we ran a series of analyses with the dependent variables (A) gestational age at first visit, which impacts whether or not a woman will become eligible for repeat HIV testing, (B) whether or not the woman returned to clinic, which impacts being offered a repeat HIV test and (C) whether or not the woman received a retest. Both bivariate and multivariable models were presented since the unadjusted models provide information on the target population for implementation of strategies, while the adjusted models account for confounding to better describe what factors drive the trends.

## 3 Results

### 3.1 Characteristics of the Study Population

A total of 2160 women who had an LMP in the years 2011, 2012 and 2013 attended antenatal clinic at the study facility. Ninety‐six women had two pregnancies fall within the time span of data collected; all women were kept in the sample but only the first pregnancy was included in analyses. We excluded 15 women from the dataset for having their first antenatal visit at a different clinic, leaving us with a sample of 2145 (Table 1). Of the 2145 women remaining in the sample, the mean age was 23.5 years (standard deviation (SD) 5.48) with a mean estimated village distance from clinic of 4.7 km (SD 6.45), with a minority of 5% living over 15 km away. Fifteen per cent of the women were either single or no longer in a marital relationship, 28.4% of all women had four or more children, and most women (80.1%) came for their first ANC visit during the second trimester, with a mean gestational age of 21.6 weeks (SD 6.3). Just over a quarter (27.5%) of all women had at least the four recommended ANC visits (30.0% when considering only women who came prior to their third trimester; and 54.7% when considering only women who had an initial visit during their first trimester).

**Table 1:** Descriptive characteristics of the study sample (N = 2145)

Variable	Mean (SD)	n (%)
Age (years)	23.5 (5.48)	
Estimated village distance from hospital (km)	4.7 (6.45)	
Gestational at first ANC visit (weeks)	21.6 (6.3)	
Year of pregnancy		
2011		519 (24.2)
2012		738 (34.4)
2013		888 (41.4)
Number of ANC visits		
1		501 (23.4)
2		509 (23.7)
3		545 (25.4)
≥4		590 (27.5)
Marital status		
Married		1785 (83.3)
Single		225 (10.4)
Widowed/Divorced/Separated		99 (4.6)
Missing		36 (1.7)
Gravida		
Primigravid		602 (28.0)
2		530 (24.7)
3		401 (18.9)
4		289 (13.5)
5		182 (8.5)
≥6		141 (6.4)
Gestational age at first ANC visit		
≤12 weeks		221 (10.3)
13 to 20 weeks		731 (34.1)
21 to 28 weeks		986 (46.0)
29 to 36 weeks		203 (9.5)
≥37 weeks		4 (0.1)
HIV status at delivery		
Known HIV positive at first visit		413 (19.3)
Newly diagnosed HIV positive in ANC		143 (6.7)
Negative		300 (14.0)
Previously negative^a^		1245 (58.0)
Not done/Missing		44 (2.0)

ANC, antenatal care.

a Defined as having had more than three months pass since the last HIV‐negative test result.

Nineteen per cent of the women already knew of their HIV‐positive status prior to their first ANC visit, while another 6.7% were diagnosed as being HIV positive for the first time at their first ANC visit of the current pregnancy. At delivery, 14.0% were considered to be known HIV negative, having had their most recent HIV test within the last three months, and 58.0% of all women went to delivery without a repeat HIV test despite having last tested HIV negative more than three months prior. Overall acceptance of the HIV test during pregnancy was very high at 98.0%.

### 3.2 Characterizing Missed Opportunities

**Figure 1:**
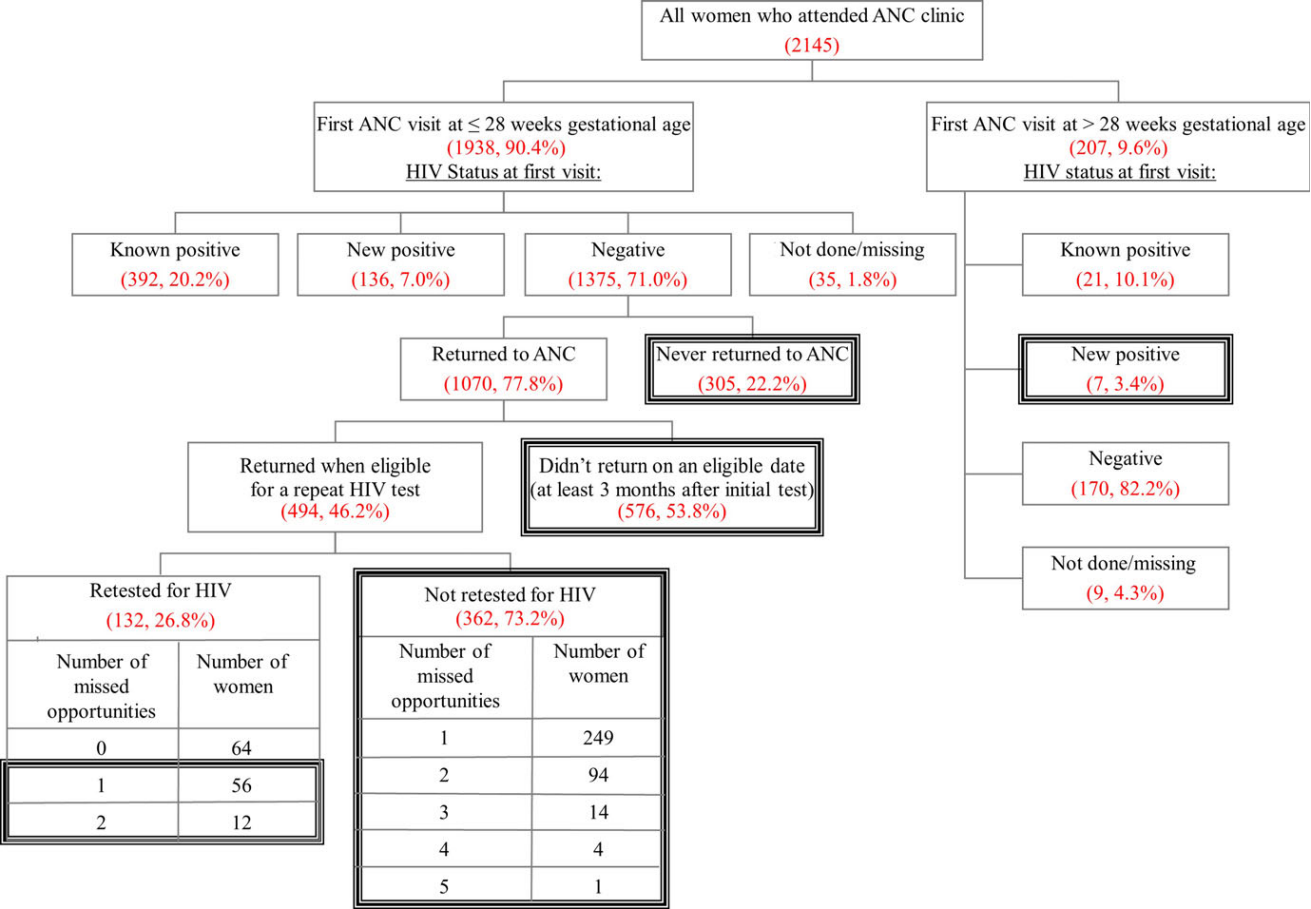
Missed opportunities for repeat HIV testing and early antiretroviral therapy initiation at a district hospital in southwestern Kenya, 2011 to 2014. Dark black boxes indicate missed opportunities for repeat HIV testing (among women whose first antenatal care (ANC) visit was at ≤28 weeks gestational age) or early intervention of mother‐to‐child transmission of HIV through initiating antiretroviral therapy (among women whose first ANC visit was >28 weeks gestational age). Among patients who returned when eligible for a repeat HIV test (n = 494), “number of missed opportunities” indicates the number of times that they came for a clinic visit when eligible for a repeat HIV test, but were not tested. Thus, among eligible returnees who were eventually retested (n = 132), some had come for one or two visits when eligible without getting a retest. Similarly, among eligible returnees who were never retested (n = 362), they may have come to ANC anywhere between one and five times when eligible without getting a retest. Percentages are a subset of the level right above.

Of all women in our sample, 207/2145 (9.6%) presented after 28 weeks gestational age for their first antenatal visit, making them ineligible for repeat HIV testing later in pregnancy or at delivery (Figure 1). Among these women, 7/207 (3.4%) tested HIV positive for the first time, possibly constituting a missed opportunity for early maternal ART initiation to suppress viral load and prevent MTCT.

The majority of women (1938/2145, 90.4%) initially presented to ANC by 28 weeks gestational age. Of those, 392/1938 (20.2%) were known HIV positive, 136/1938 (7.0%) were newly HIV positive, and 35/1938 (1.8%) had missing test results, leaving 1375/1938 (71.0%) women who tested HIV negative eventually eligible for a retest later in pregnancy. Of these eligible women, 305/1375 (22.2%) never returned to the ANC clinic and 576/1070 (53.8%) returned to clinic but not on a date at which they were eligible for retesting, since they came before 12 weeks had passed. Of the 494/1070 (46.2%) who returned when eligible, 132/494 (26.8%) were eventually retested, while 362/494 (73.2%) were not retested, even though eligible at one or more visits. Of the total of 1375 women who should have had a repeat HIV test later in pregnancy, only 132 (9.6%) were retested.

Thus, missed opportunities to retest the target population of 1375 potentially eligible women included (A) women who did not return to clinic at all, (B) women who did not have visits spaced out in such a way that they were eligible when they did return, and (C) women who were not retested, although eligible at one or more ANC visits.

### 3.3 Characterizing factors associated with process and outcome measures

#### 3.3.1 Factors associated with early gestational age at first visit

Having a first ANC visit early in pregnancy impacts eligibility for HIV retesting (as per the 28 weeks gestational age threshold) and allows for more time to fit in the four ANC visits recommended by the Ministry of Health. In bivariate analyses, known HIV‐positive status prior to first visit was significantly associated with earlier gestational age at first visit when compared with being HIV negative or newly diagnosed as HIV positive (Table 2). Later year of pregnancy and older age were similarly associated with earlier gestational age at first visit, a trend that held true even when we dichotomized the gestational age to being less than/equal to or greater than 28 weeks at first ANC visit (data not shown). Conversely, being unmarried or previously married (widowed, divorced, or separated) was associated with later average gestational age at first visit when compared with being married. In multivariable analyses, after adjusting for the effect of HIV status at first visit, marital status, year of pregnancy, and village distance from clinic, older age was no longer a significant predictor of gestational age at first visit.

**Table 2:** Factors affecting gestational age at first visit and return to clinic

		Gestational age at first visit (weeks)				Returned to clinic at least once			
		Bivariate analyses		Multivariable analyses		Bivariate analyses		Multivariable analyses	
Factor	N	Mean gestational age (SD)	*p*‐value	β (SE)	*p*‐value	OR (95% CI)	*p*‐value	aOR (95% CI)	*p*‐value
HIV status at first visit									
Negative	1545	22.4 (6.0)	<0.0001	Ref.	<0.0001	Ref.	<0.0001	Ref.	<0.001
Newly positive	143	21.4 (5.7)		−0.80 (0.55)		0.74 (0.51, 1.07)		0.62 (0.42, 0.92)	
Known positive	413	18.6 (6.5)		−3.60 (0.36)		2.38 (1.69, 3.14)		1.85 (1.32, 2.58)	
Marital status									
Married	1785	21.3 (6.4)	<0.0001	Ref.	<0.0001	Ref.	<0.0001	Ref.	<0.001
Prev. married	99	22.7 (5.3)		0.70 (0.66)		0.55 (0.35, 0.85)		0.56 (0.35, 0.90)	
Unmarried	225	23.8 (5.5)		2.07 (0.47)		0.38 (0.29, 0.52)		0.49 (0.35, 0.69)	
LMP year^b^									
2011	519	22.3 (5.9)	<0.05	Ref.	<0.05	Ref.	<0.05	Ref.	0.159
2012	738	21.7 (6.3)		−0.81 (0.36)		0.92 (0.70, 1.22)		0.92 (0.68, 1.23)	
2013	888	21.2 (6.4)		−1.29 (0.35)		0.74 (0.57, 0.96)		0.77 (0.58, 1.02)	
Age of patient^b^									
≤15 years	124	23.4 (6.0)	<0.0001	Ref.	0.437	Ref.	<0.0001	Ref.	0.052
16 to 20 years	649	22.4 (5.9)		−0.49 (0.63)		1.18 (0.78, 1.78)		1.06 (0.68, 1.65)	
21 to 30 years	1147	21.2 (6.4)		−0.28 (0.63)		1.89 (1.27, 2.8)		1.33 (0.84, 2.10)	
≥31 years	225	20.8 (6.8)		0.04 (0.75)		2.97 (1.75, 5.05)		1.87 (1.04, 3.39)	
Village distance from clinic^b^									
≤5 km	1598	21.7 (6.3)	<0.05	Ref	0.150	Ref.	0.768	Ref.	0.714
6 to 15 km	334	21.9 (6.1)		0.52 (0.37)		1.09 (0.82, 1.45)		1.07 (0.79, 1.44)	
16 to 30 km	93	18.8 (6.3)		−1.55 (0.66)		0.99 (0.60, 1.61)		0.78 (0.46, 1.34)	
≥31 km	21	18.1 (5.4)		−2.36 (1.34)		0.98 (0.35, 2.69)		0.74 (0.26, 2.12)	
Parity^a^,^b^									
Primigravid	602	21.5 (6.0)	0.441	N/A	N/A	Ref.	<0.0001	N/A	N/A
2	530	21.7 (6.4)		N/A		1.4 (1.07, 1.84)		N/A	
3	401	21.2 (6.5)		N/A		1.59 (1.18, 2.15)		N/A	
4	608	21.9 (6.2)		N/A		1.74 (1.33, 2.28)		N/A	

CI, Confidence interval; SD, standard deviation; SE, standard error; β, regression beta coefficient; LMP, last menstrual period; Prev., previously; N/A, not applicable; OR, odds ratio; aOR, adjusted odds ratio; Ref., reference.

a Multivariable analyses controlled for all other variables in the table except for parity because of its collinearity with age.

b Reported *p*‐values are for trend.

Unexpectedly, in bivariate analyses, women who lived at a greater distance from the clinic came, on average, earlier than women who lived closer to the clinic. To assess this finding, we ran an analysis and determined that being of known HIV‐positive status and older age correlated with living further away, but marital status did not (data not shown). In multivariable analyses, after adjusting for both HIV status and age, characteristics that are significant predictors of gestational age of first visit, travelling a greater distance to clinic was no longer associated with earlier gestational age at first visit (Table 2).

Sensitivity analyses excluding the small population who lived far away from the clinic (operationalised as the 5% of the population who lived in a village ≥15 km away and who appeared to have different characteristics than other clients) did not qualitatively change the reported results.

#### 3.3.2 Factors associated with returning to clinic

Returning to clinic is a prerequisite for getting retested for HIV. Having a first ANC visit by 28 weeks gestational age was associated with 3.75 times the odds (95% CI: 2.79, 5.05) of returning to clinic over having a first ANC visit between 29 to 36 weeks. In bivariate analyses, known HIV‐positive status prior to first visit was associated with 2.38 times the odds (95% CI: 1.69, 3.14) of returning to clinic compared with being HIV negative (Table 2).

In multivariable analyses, women who were of known HIV‐positive status, were married, and/or were age 31 and older were significantly more likely to return to clinic. Being diagnosed with HIV during pregnancy was also a risk factor for failing to return to antenatal clinic, as newly positive women had a 0.62 odds (95% CI: 0.42, 0.92) of returning to clinic compared to those who tested negative in pregnancy even after adjusting for potential confounders. This is in contrast to women who knew their HIV‐positive status prior to initiating ANC and who had significantly increased odds of returning to clinic. Being unmarried and aged 20 or younger was associated with an increase in mean gestational age of first visit by 2.52 weeks (95% CI: 1.56, 3.48) and a 2.59 odds (95% CI: 1.90, 3.54) of failing to return to clinic, compared to those who were married and over 20 years of age.

#### 3.3.3 Factors associated with getting retested for HIV

In our target population, all the women who came early enough to be eligible for retesting (n = 1375), we assessed the relationship between patient characteristics and retesting. In bivariate analyses, we found that only marital status and year of pregnancy were significantly associated with getting retested (Table 3).

**Table 3:** Factors associated with getting retested for HIV (bivariate analyses)

	Retested for HIV among target population (n = 1375)				Retested for HIV among eligible women who returned to clinic (n = 494)			
Factor	N	Odds Ratio	(95% CI)	*p*‐value	N	Odds Ratio	(95% CI)	*p*‐value
Marital status								
Married	1113	Ref.		0.038	425	Ref.		0.73
Unmarried or previously married	237	0.54	(0.30, 0.96)		56	0.89	(0.47, 1.7)	
LMP year								
2011	316	Ref.		<0.0001	105	Ref.		<0.0001
2012	466	22.2	(3.01, 163)		162	24.3	(3.27, 181)	
2013	593	63.4	(8.80, 456)		227	81.0	(11.1, 591)	
Age of patient								
≤15 years	89	Ref.		0.17	21	Ref.		0.52
16 –20 years	479	2.21	(0.77, 6.33)		157	0.99	(0.23, 3.84)	
21–30 years	711	2.58	(0.92, 7.24)		283	1.72	(0.54, 5.40)	
≥31 years	96	1.41	(0.38, 5.19)		33	1.58	(0.51, 4.87)	
Parity								
Primigravid	479	Ref.		0.77	21	Ref.		0.91
2	346	1.09	(0.69, 1.73)		157	0.97	(0.58, 1.60)	
3	247	0.93	(0.55, 1.58)		283	0.97	(9.54, 1.75)	
4	301	0.83	(0.50, 1.38)		33	0.82	(0.46, 1.44)	
Village distance from clinic								
≤15 km	1264	Ref.		0.17	457	Ref.		0.11
>15 km	50	0.37	(0.09, 1.5)		19	0.30	(0.70, 1.35)	

CI, Confidence interval; LMP, last menstrual period; Ref., reference; km, kilometers

When considering the subset of eligible women who did return to clinic (n = 494), we found that only year of pregnancy was associated with getting retested. Using a multivariable Poisson regression model to examine the factors associated with having multiple missed opportunities for retesting yielded a similar result. Of these 494 women, we found that in 2011, only 1 of 105 eligible patients was retested (0.95%); in 2012, 31 of 162 eligible patients were retested (19.14%) and in 2013, 100 of 227 (44.05%) eligible patients were retested.

**Figure 2:**
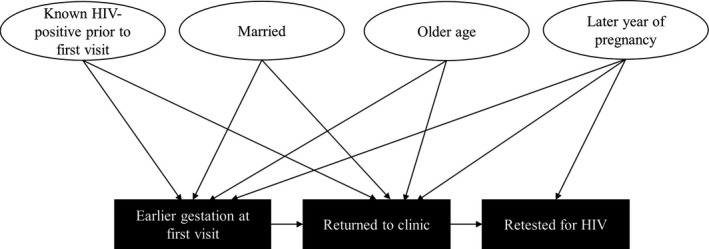
Factors associated with earlier gestational age at first visit, returning to clinic, and getting retested for HIV (bivariate analyses). Arrows indicate a statistically significant positive association between pregnant women's characteristics and process measures (earlier gestation at first visit and return to clinic) as well as the outcome measure (getting retested for HIV).

The results of bivariate analyses from Tables 2 and 3 are summarized in Figure 2 to highlight the target population for implementation of strategies. The presence of an arrow indicates a statistically significant positive association.

### 3.4 Outcomes of repeat HIV testing

The 132 women who were retested contributed a mean of 125 days (range = 83 to 196 days) between initial HIV test and retest, for a total of 45.4 person‐years. Two women seroconverted from being HIV negative at initial test to being HIV positive on retest, corresponding to a (2/132) 1.5% cumulative incidence and an incidence rate of (100/45.4×2) 4.4 per 100 person‐years. Extrapolating the incidence rates to our target population would suggest that had we retested all potentially eligible women (n = 1375), we may have identified an additional 18 women who had seroconverted by delivery.

## 4 Discussion

Repeat HIV testing and early ART initiation are important components of ANC in settings of high HIV incidence. Utilizing routinely collected antenatal record data from a large hospital in southwestern Kenya, our study found that the dissemination and implementation of guidelines for repeat HIV testing, which occurred in 2012, were successful in making a considerable impact on rates of repeat HIV testing through encouraging provider‐initiated rather than patient‐initiated requests for retesting. Specifically, guideline dissemination was associated with an increase in retesting from less than 1% among women with LMPs in 2011 to nearly 45% among women with LMPs in 2013. However, missed opportunities continue to exist for both repeat HIV testing and early ART initiation, leading to potential MTCT of HIV that may have otherwise been intervened upon.

Key missed opportunities included later gestational age at first ANC visit, leading to potentially delayed initial identification of HIV seropositivity and linkage to HIV care, failing to return to antenatal clinic after an initial visit, and failing to get retested even though eligible. Conversely, earlier gestational age was positively associated with returning to clinic, which was in turn a prerequisite for getting retested for HIV. Factors contributing to earlier gestational age at first visit included known HIV‐positive status prior to first visit, being married, and being of older age. Similar factors contributed to likelihood of returning to clinic. Thus, all these demographic characteristics may have influenced likelihood of getting retested for HIV.

We found that initial HIV testing rates (98.0%) in our study were higher than the national average (93.1%), potentially due to a higher HIV prevalence in the region 9. In a recent meta‐analysis, Drake *et al*. reported the pooled cumulative incidence of HIV during pregnancy to be 1.5% (95% CI 1.2% to 1.8%), although African countries had a higher rate when compared to non‐African countries (3.6% vs. 0.3%, respectively, *p *<* *0.001) 18. They also reported a pooled incidence rate of 4.7 (95% CI 3.3 to 6.1) per 100 person‐years 18. Kinuthia *et al*. reported in 2010 data from the Nairobi and Nyanza regions of Kenya supporting a cumulative incidence 2.6% and an incidence rate of 6.8 per 100 person‐years 12. The cumulative incidence rate of 1.5% and incidence rate of 4.4 per 100 person‐years reported in this study thus correspond closely to the rates reported in the literature. Our incidence rates should be interpreted in the light of the fact that they were estimated among women who returned to ANC when eligible and received a retest, a group that may have a different risk profile for HIV acquisition during pregnancy than women who did not return to ANC or who were not retested for other reasons. We also found that known HIV‐positive status prior to first visit was significantly associated with earlier gestational age at first visit and returning to clinic, possibly because this group is already engaged in HIV care services at the facility, is used to accessing care, or have been encouraged by HIV providers to access ANC early to prevent MTCT.

Several themes relevant to improving the implementation of repeat HIV testing guidelines emerged from the data. At the patient level, various types of stigmas may have influenced ANC choices. For women who were young and/or unmarried, as well as women who were previously married, stigma may have influenced a later average gestational age at first presentation to clinic, as well as a lower likelihood of returning to clinic and thus lower retesting rates. These data are corroborated by both the qualitative 19 and quantitative 20 literature. We also found that women newly diagnosed as being HIV positive in ANC were significantly less likely to return to clinic, a crucial group to focus on for the prevention of MTCT and linkage to care for their own health. Women who were known HIV positive were more likely to live further away from the clinic, suggesting that stigma may have led them to seek ANC away from their home, just as the literature indicates that HIV stigma may lead individuals to seek general HIV care far from home 21.

At the clinic level, factors unrelated to patient choices may have been more influential in determining who among eligible returnees received retesting, as seen by the fact that receipt of retesting was uncorrelated with demographic characteristics. This also suggests that providers did not seem to target certain profiles of women for retesting. In contrast, year of LMP among eligible returnees was highly predictive of getting retested, indicating that the dissemination of retesting guidelines may have driven an increase in provider‐initiated testing. However, the fact that over 55% of women still failed to get retested more than a year after guideline dissemination (since women with LMPs in late 2013 delivered as late as October 2014) and that women continued to fall through the cracks even though eligible at multiple visits is concerning. Our prior study found that ability of providers to remember when three months have elapsed since last HIV test, clinic workload on day of patient visit, and availability of adequate HIV test kits may impact whether providers offer retesting 19.

This study has several strengths including the longitudinal nature of the data which contained both demographic factors and process/outcome measures. It also spanned the pregnancy duration as well as the time prior to and after the dissemination of national repeat HIV testing guidelines. We were limited by our inability to determine if women were retested at some point during their pregnancy at other ANC facilities, although we mitigated this likelihood by restricting the dataset to women who had their first ANC visit at our site and thus likely treated it as their primary care location. Similarly, we were unable to determine if miscarriage was a reason for non‐return to ANC and were limited in our sociodemographic variables to those which are routinely collected data. This was also a single, semi‐rural study site that was supported by FACES and thus may not be representative of ANCs in other areas. Finally, we were unable to assess whether HIV retesting occurred at delivery and/or the postnatal periods, both of which would include periods of continued high risk of HIV acquisition and transmission. In spite of these limitations and the age of the data at time of publication, we believe our findings are applicable to many settings around the globe with continuing high MTCT incidence. This is because we (a) provide a framework for conceptualizing missed opportunities early in the HIV care cascade at the point of identifying pregnant women who are newly HIV positive despite having recently tested HIV negative, and (b) highlight vulnerable groups that may be at higher risk of incident HIV infection and MTCT but fail to get retested.

## 5 Conclusions

In conclusion, repeat HIV testing rates seem to have increased in the post‐guideline period, but improving late‐pregnancy detection of incident HIV infection may require community mobilization and messaging surrounding earlier and more consistent ANC visits, strategies that will also likely improve general ANC, and the importance of HIV retesting. This is particularly significant in the current era of initiating immediate, lifelong ART for all pregnant women who are living with HIV. Further research should assess whether these findings are applicable to other settings, determine the driving factors for multiple missed opportunities for eligible women, and assess clinic‐level factors such time of day or day of week that may impact provider‐initiated testing. In addition, other studies should attempt to link ANC testing with delivery and postnatal testing to assess retesting and ART initiation and retention for women that acquired HIV in the perinatal period.

## Competing interests

The authors have no competing interests to declare.

## References

[1] World Health Organization . Consolidated guidelines on HIV testing services. 5Cs: consent, confidentiality, counselling, correct results and connection. Geneva, Switzerland: WHO Press; 2015. 26378328

[2] World Health Organization, HIV/AIDS Programme . Programmatic update: Use of antiretroviral drugs for treating pregnant women and preventing HIV infection in infants. Executive summary. World Health Organization[cited 2017 Oct 12]. Available from: http://www.who.int/hiv/PMTCT_update.pdf

[3] Johnson LF , Stinson K , Newell ML , Bland RM , Moultrie H , Davies MA , et al. The contribution of maternal HIV seroconversion during late pregnancy and breastfeeding to mother‐to‐child transmission of HIV. J Acquir Immune Defic Syndr. 2012;59(4):417–25. 22193774 10.1097/QAI.0b013e3182432f27 PMC3378499

[4] Lehman DA , Farquhar C . Biological mechanisms of vertical human immunodeficiency virus (HIV‐1) transmission. Rev Med Virol. 2007;17(6):381–403. 17542053 10.1002/rmv.543

[5] Garcia PM , Kalish LA , Pitt J , Minkoff H , Quinn TC , Burchett SK , et al. Maternal levels of plasma human immunodeficiency virus type 1 RNA and the risk of perinatal transmission. Women and Infants Transmission Study Group. N Engl J Med. 1999;341(6):394–402. 10432324 10.1056/NEJM199908053410602

[6] World Health Organization (WHO) . What's new in infant HIV diagnosis. Fact sheet: HIV treatment and care. 2015.

[7] World Health Organization HIV/AIDS Programme . WHO recommendations on the diagnosis of HIV infection in infants and children. Geneva: World Health Organization; 2010. 23741779

[8] Kenya Ministry of Health . EMTCT Strategic Framework: Towards the elimination of mother to child transmission of HIV and keeping mothers alive. Nairobi, Kenya; 2012.

[9] Sirengo M , Muthoni L , Kellogg TA , AA Kim , Katana A , Mwanyumba S , et al. Mother‐to‐child transmission of HIV in Kenya: results from a nationally representative study. J Acquir Immune Defic Syndr. 2014;66:S66–74. 24732822 10.1097/QAI.0000000000000115 PMC4790087

[10] Ng'eno B , Mwangi A , Ng'ang'a L , Kim AA , Waruru A , Mukui I , et al. Burden of HIV infection among children aged 18 months to 14 years in kenya: results from a nationally representative population‐based cross‐sectional survey. JAIDS Journal of Acquired Immune Deficiency Syndromes. 2014;66:S82–8. 24732823 10.1097/QAI.0000000000000118 PMC4784690

[11] Republic of Kenya Ministry of Health . National AIDS and STI Control Programme. Guidelines for prevention of mother to child transmission (PMTCT) of HIV/AIDS in Kenya. Nairobi, Kenya; 2012.

[12] Kinuthia J , Kiarie JN , Farquhar C , Richardson B , Nduati R , Mbori‐Ngacha D , et al. Cofactors for HIV‐1 incidence during pregnancy and postpartum period. Curr HIV Res. 2010;8(7):510–4. 20946093 10.2174/157016210793499213 PMC3372399

[13] Heemelaar S , Habets N , Makukula Z , van Roosmalen J , van den Akker T . Repeat HIV testing during pregnancy and delivery: missed opportunities in a rural district hospital in Zambia. Trop Med Int Health. 2015;20(3):277–83. 25418130 10.1111/tmi.12432

[14] Kimanga DO , Ogola S , Umuro M , Ng'ang'a A , Kimondo L , Murithi P , et al. Prevalence and incidence of HIV infection, trends, and risk factors among persons aged 15‐64 years in Kenya: results from a nationally representative study. J Acquir Immune Defic Syndr. 2014;66:S13–26. 24445338 10.1097/QAI.0000000000000124 PMC4794992

[15] Maina WK , Kim AA , Rutherford GW , Harper M , K'Oyugi BO , Sharif S , et al. Kenya AIDS indicator surveys 2007 and 2012: implications for public health policies for HIV prevention and treatment. J Acquir Immune Defic Syndr. 2014;66:S130–7. 24732817 10.1097/QAI.0000000000000123 PMC4784700

[16] Lewis Kulzer J , Penner JA , Marima R , Oyaro P , Oyanga AO , Shade SB , et al. Family model of HIV care and treatment: a retrospective study in Kenya. J Intern AIDS Soc. 2012;15(1):8. 10.1186/1758-2652-15-8 PMC3298805 22353553

[17] Agency for Healthcare Research and Quality (AHRQ) . Selecting process measures for clinical quality measurement. 2016 [cited 2017 Oct 12] Available from: https://www.qualitymeasures.ahrq.gov/help-and-about/quality-measure-tutorials/selecting-process-measures

[18] Drake A , Wagner A , Richardson B , John‐Stewart G . Incident HIV during pregnancy and postpartum and risk of mother‐to‐child HIV transmission: a systematic review and meta‐analysis. PLoS Med. 2014;11(2):e1001608. 24586123 10.1371/journal.pmed.1001608 PMC3934828

[19] Rogers AJ , Weke E , Kwena Z , Bukusi EA , Oyaro P , Cohen CR , et al. Implementation of repeat HIV testing during pregnancy in Kenya: a qualitative study. BMC Pregnancy Childbirth. 2016;16(1):151. 27401819 10.1186/s12884-016-0936-6 PMC4940827

[20] Ronen K , McGrath CJ , Langat AC , Kinuthia J , Omolo D , Singa B , et al. Gaps in adolescent engagement in antenatal care and prevention of mother‐to‐child HIV transmission services in Kenya. J Acquir Immune Defic Syndr. 2017;74(1):30–7. 27599005 10.1097/QAI.0000000000001176 PMC5895459

[21] Tomori C , Kennedy CE , Brahmbhatt H , Wagman JA , Mbwambo JK , Likindikoki S , et al. Barriers and facilitators of retention in HIV care and treatment services in Iringa, Tanzania: the importance of socioeconomic and sociocultural factors. AIDS Care. 2014;26(7):907–13. 24279762 10.1080/09540121.2013.861574

